# Gastroduodenal Artery Pseudoaneurysm Causing Upper Gastrointestinal Bleeding

**DOI:** 10.7759/cureus.87799

**Published:** 2025-07-12

**Authors:** Nadine Abul-Khoudoud, Jack Ludwig, Ravi Patel, Kulveer Dabb, Joseph F Staffetti

**Affiliations:** 1 Internal Medicine, HCA Healthcare/University of South Florida (USF) Morsani College of Medicine, Regional Medical Center Bayonet Point, Hudson, USA; 2 Gastroenterology, HCA Healthcare/University of South Florida (USF) Morsani College of Medicine, Regional Medical Center Bayonet Point, Hudson, USA

**Keywords:** artery, bleed, egd, embolization, gastroduodenal, gastrointestinal, microcoil, non-steroidal anti-inflammatory drug, pseudoaneurysm

## Abstract

Gastroduodenal artery pseudoaneurysms (GDAPs) are a rare but potentially life-threatening condition characterized by the formation of a blood-filled sac secondary to disruption of the arterial wall, frequently resulting from trauma, pancreatitis, or ulceration. We present the case of a 73-year-old female diagnosed with a GDAP of unknown etiology. Management included collaboration with the interventional radiology team for angiography and coil embolization. This case highlights the importance of early recognition and multidisciplinary management, including imaging, endovascular intervention, and surgical consideration.

## Introduction

Gastroduodenal artery pseudoaneurysms (GDAPs) are uncommon vascular lesions that are often difficult to diagnose and can lead to significant hemorrhage. These pseudoaneurysms represent roughly 1.5% of all reported visceral artery aneurysms and can lead to life-threatening complications such as rupture and bleeding [1-3]. The abdominal aorta supplies the splanchnic circulation through three main branches: the celiac trunk, the superior mesenteric artery, and the inferior mesenteric artery. The celiac trunk arises just below the diaphragm at the T12 vertebral level and quickly divides into the left gastric, splenic, and common hepatic arteries. The common hepatic artery further branches into the proper hepatic artery and the gastroduodenal artery (GDA). The GDA supplies the stomach, duodenum, and parts of the pancreas, and can develop a pseudoaneurysm following trauma, inflammation (e.g., pancreatitis), or peptic ulcer disease [1,4,5]. Due to the risk of severe hemorrhage, early identification and prompt management are critical. Endovascular management has become the gold standard intervention for these pseudoaneurysms; however, surgery may be required for more complex cases [4,6]. This case demonstrates the multidisciplinary management of a GDAP, which resulted in a successful outcome.

## Case presentation

The patient is a 73-year-old female with a past medical history significant for gastroesophageal reflux disease (GERD)/gastritis, hypertension, hyperlipidemia, and osteoarthritis with daily non-steroidal anti-inflammatory drug (NSAID) use for years, who was brought to the emergency department by emergency medical services (EMS) after being found on the floor in the bathroom in a pool of bright red blood by a family member. The patient became too weak to stand and lowered herself to the floor due to lightheadedness and dizziness. She subsequently passed a large-volume, bright red stool. EMS confirmed the volume of blood was significant. Additionally, she had several episodes of vomiting bright red blood. She denied abdominal pain, prior gastrointestinal bleeding, or a history of peptic ulcer disease. The patient did report a screening colonoscopy four years prior, which revealed only benign polyps.

Initial vital signs were concerning for hemorrhagic shock, with a blood pressure of 84/53 mmHg and tachycardia at 137 beats per minute. Laboratory tests revealed a hemoglobin level of 9.5 g/dL, which had significantly decreased from the most recent available level of 14.0 g/dL three years earlier. On exam, the patient was fatigued but alert and oriented to person, place, and time. She had dried blood in her nares and around her mouth. Her abdomen was soft, non-tender, and non-distended. The patient was hemodynamically stabilized with one liter of intravenous fluid and transfusion of two units of packed red blood cells.

**Figure 1:**
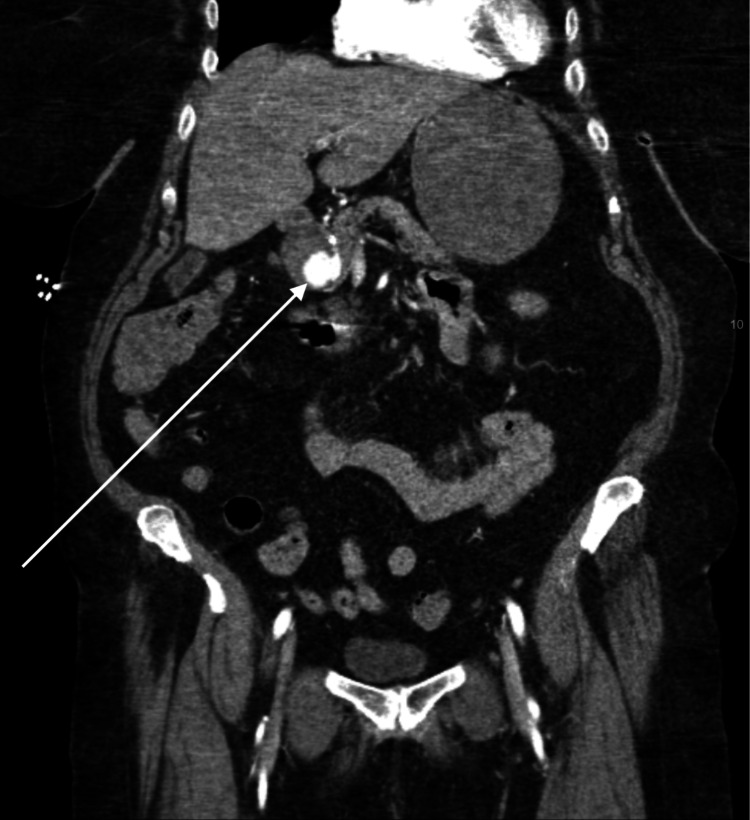
CTA (computed tomography angiogram) of abdomen and pelvis with extravasation from a branch of the celiac artery into a cystic space, found to be a large gastroduodenal artery pseudoaneurysm.

**Figure 2:**
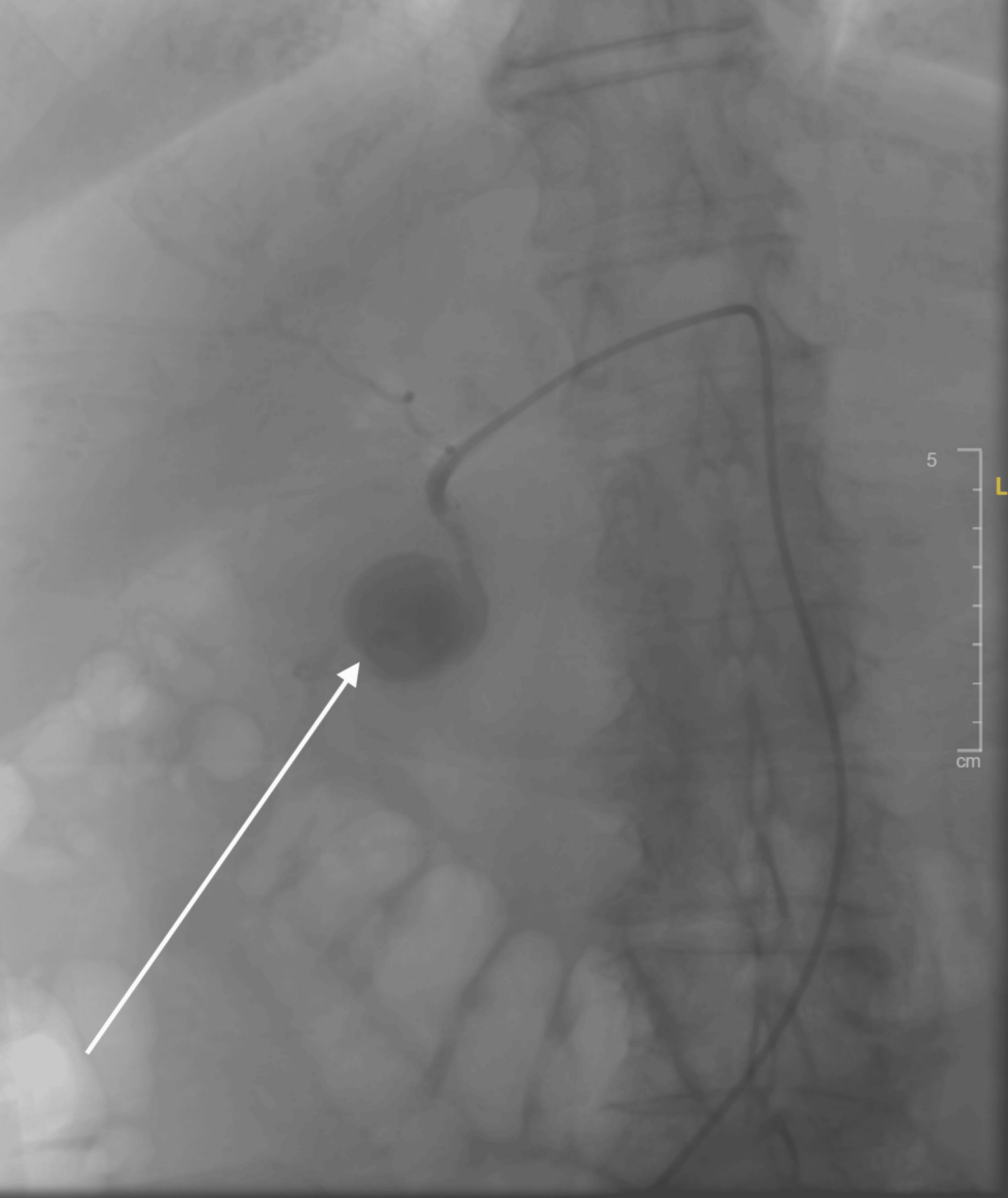
Aortography revealing the gastroduodenal artery pseudoaneurysm, measuring 6.7 x 4.7 x 4.8 cm.

**Figure 3:**
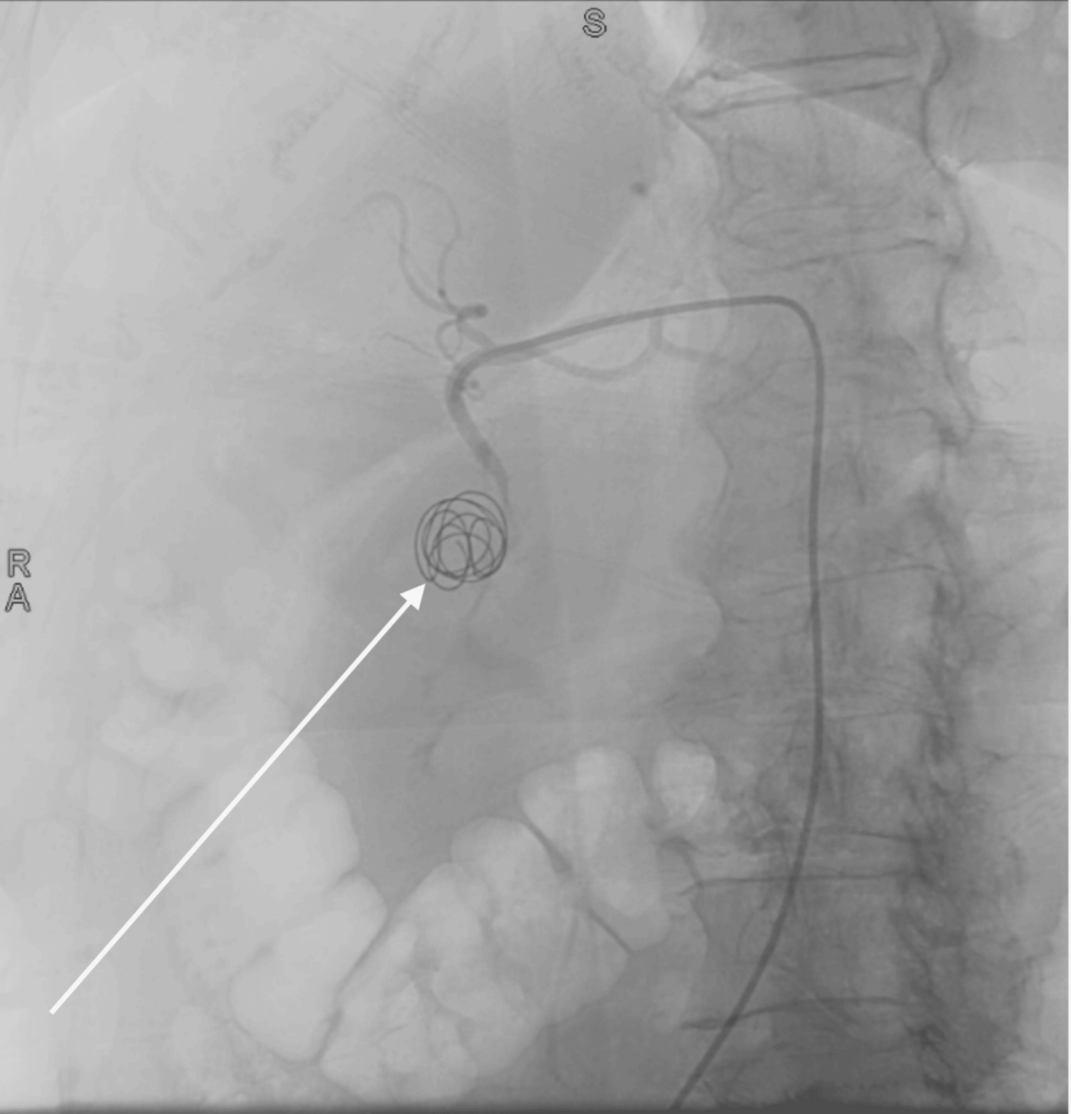
Aortography of the gastroduodenal pseudoaneurysm after microcoil embolization by interventional radiology.

A computed tomography angiogram (CTA) of the abdomen and pelvis revealed extravasation from a branch of the celiac artery into a cystic space, thought to represent a large pseudoaneurysm (see Figure 1). However, bleeding into a pseudocyst or a duodenal diverticulum was also considered in the differential. Given the location and size of the pseudoaneurysm, the patient was transferred to the interventional radiology suite for endovascular embolization. Superselective gastroduodenal angiography demonstrated a large pseudoaneurysm (6.7 × 4.7 × 4.8 cm) arising from the gastroduodenal artery, without a breach in the lumen (see Figure 2). A catheter was advanced into the gastroduodenal artery, and coil embolization was performed successfully, with complete occlusion of the pseudoaneurysm on follow-up angiography (see Figure 3). The procedure was well tolerated, and no complications were observed.

Post-procedure, the patient's vital signs stabilized, and she was closely monitored in the intensive care unit. Her hemoglobin level rose to 11.1 g/dL following the initial transfusion and embolization. The patient experienced two episodes of melena the day after the procedure, accompanied by a drop in hemoglobin to 8.0 g/dL. One additional unit of packed red blood cells was transfused, and follow-up labs revealed a hemoglobin level of 9.1 g/dL, with only mild fluctuations on continued monitoring for the remainder of her admission. Esophagogastroduodenoscopy (EGD) was not performed after the GDAP embolization due to concern for potentially worsening the treated bleed with instrumentation. The patient was discharged on day five of hospitalization with strict recommendations for a follow-up EGD within two to four weeks to monitor for the potential development of a penetrating ulcer at the level of the treated pseudoaneurysm. She was prescribed an eight-week course of an oral proton-pump inhibitor (PPI) and instructed to avoid all NSAIDs. The patient underwent outpatient upper and lower endoscopy two months later. EGD revealed a small hiatal hernia, and colonoscopy showed multiple polyps, a few diverticula in the sigmoid colon, and small internal hemorrhoids.

## Discussion

Pseudoaneurysms occur most commonly in the fifth decade of life, have a mean size of 3.6 cm, and exhibit a male-to-female ratio of 4.5:1. They most frequently involve the splenic artery (46%), renal artery (22%), hepatic artery (16.2%), and gastroduodenal artery (1.5%) [3,7]. In this case, the specific etiology of the patient’s GDAP remains unclear. However, common precursors to these pseudoaneurysms include peptic ulcer disease, pancreatitis or pseudocysts, abdominal trauma, surgical or endoscopic complications, and, less commonly, vascular anatomic variants [8]. A similar case reported by Boparai et al. described a patient with a GDA pseudoaneurysm who presented with hematemesis and acute blood loss anemia. Like our patient, they experienced gastrointestinal hemorrhage requiring blood transfusion and emergent coil embolization of the gastroduodenal artery. The patient had a history of daily ibuprofen use, which was believed to have caused a large duodenal ulcer, seen on upper endoscopy, that eroded into the gastroduodenal artery [9]. GDAPs are rare but pose significant risk due to the potential for rupture and severe hemorrhage. The mortality rate in cases of ruptured GDAP is approximately 40%, underscoring the need for early recognition and intervention to avoid fatal complications. Common clinical presentations include abdominal pain, melena, hematemesis, obstruction of surrounding structures, and hemorrhagic shock [7,10,11]. Diagnosis is frequently achieved using cross-sectional imaging, with computed tomography angiography (CTA) of the abdomen and pelvis now considered the gold standard. CTA can reveal the characteristic features of a pseudoaneurysm, including a well-defined, blood-filled sac with a prominent vessel neck [1,4,6]. The sensitivity of CTA for detecting pseudoaneurysms is 100%, compared with 67% for conventional CT and 50% for ultrasound. Endovascular embolization has become the treatment of choice for managing GDAP, offering a less invasive alternative to surgery and yielding excellent outcomes in terms of hemorrhage control and long-term survival. Surgical intervention may be considered in cases where endovascular techniques are unsuccessful or if the patient has contraindications to embolization [4,6,11]. Interestingly, ulcers are not only a significant risk factor for GDAP but may also develop as a complication of its treatment. Microcoils used during embolization have an estimated 3% migration rate and may lead to erosion, ulceration, or rebleeding [4,12].

For this patient, in the setting of frequent NSAID use and no surgical or endoscopic history, an ulcer at the level of the pseudoaneurysm is the most probable etiology. However, an ulcer was not seen on her follow-up EGD two months later. If an ulcer was present during hospitalization, it likely healed with the oral proton pump inhibitor (PPI) prescribed upon discharge.

## Conclusions

This case report reviews the presentation and management of a 73-year-old female who presented with a massive gastrointestinal bleed and hemorrhagic shock secondary to a rare gastroduodenal artery pseudoaneurysm. This case highlights the necessity of maintaining high clinical suspicion and pursuing early intervention to avoid the life-threatening consequences of GDAP rupture. Although uncommon, GDAP should always be considered in patients presenting with severe gastrointestinal bleeding, particularly those with a history of pancreatitis or peptic ulcer disease. While this patient did not report a history of either condition, she did endorse chronic NSAID use with meloxicam, which may have contributed to the development of an ulcer at the time of presentation. Prompt imaging with CTA of the abdomen and pelvis enabled timely endovascular embolization by interventional radiology. This case demonstrates the life-threatening urgency of GDAP and the crucial role of prompt intervention.
